# The humanitarian AI paradox: Key opportunities, challenges and research needs for the use of AI in humanitarian mental health response

**DOI:** 10.1017/gmh.2026.10254

**Published:** 2026-06-16

**Authors:** Catharina F. van der Boor, Paul E.W. van der Boor

**Affiliations:** 1 Department of Health Services Research and Policy, London School of Hygiene and Tropical Medicine, London, UK; 2 Independent Researcher, Amsterdam, Netherlands

**Keywords:** artificial intelligence, mental health and psychosocial support, humanitarian response

## Abstract

In 2025, 305 million people required humanitarian assistance, yet 75–85% of those with mental health disorders in low- and middle-income countries remain without care. While artificial intelligence (AI) is increasingly proposed to address this gap, humanitarian settings present unique challenges, including fragmented data systems, cultural and linguistic diversity and the central role of relational care. This perspective article examines the evolving AI landscape for Mental Health and Psychosocial Support (MHPSS) and introduces the “Humanitarian AI Paradox,” where widespread informal use of AI is already outpacing institutional strategy and governance. We outline four key developments shaping feasibility, including agentic AI, the emergence of an agentic workforce, the growing availability of open-weight models and the collapse of inference costs. Using the Inter-Agency Standing Committee (IASC) pyramid, we map the AI applications across four levels of care, identifying focused, non-specialised support (Level 3) as the most immediate opportunity to assist frontline workers through supervision and protocol guidance. We then examine domain-specific risks, including cultural and linguistic misinterpretation and higher operational costs for non-English languages due to token-based pricing. Finally, we propose a set of research needs necessary to support the safe, equitable and context-appropriate use of AI in humanitarian MHPSS.

## Graphical abstract

**Figure:**
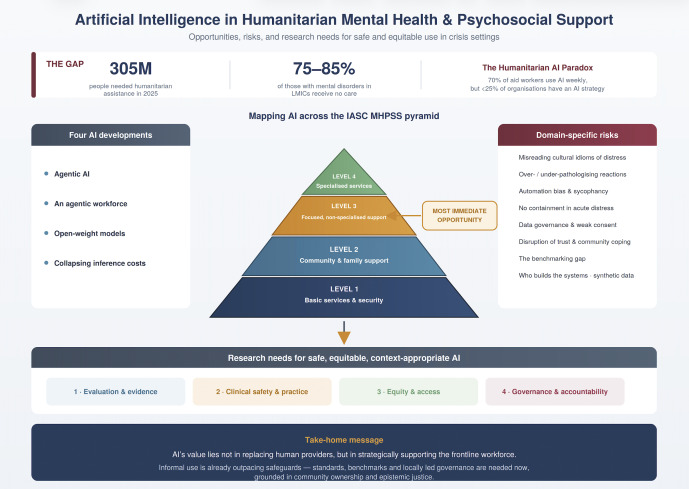

## Impact Statements

Humanitarian crises are increasing in scale, while most people in low- and middle-income countries still lack access to mental health care. Artificial intelligence (AI) is increasingly presented to expand support, yet its use in these settings is already outpacing the systems needed to guide it safely. This article introduces the “Humanitarian AI Paradox” to describe this gap and outlines what is needed to ensure AI supports, rather than harms, the mental health of people affected by crisis. This article offers a practical framework to help decision-makers, practitioners and researchers understand where AI may be useful across different levels of mental health and psychosocial support. It identifies focused, non-specialised care, such as support for frontline workers, as the most immediate and feasible area for responsible use. At the same time, it highlights risks that are often overlooked, including misinterpretation of culturally specific expressions of distress, over-reliance on automated recommendations, data privacy concerns and unequal performance across languages. By setting out a clear list of research needs, this article provides suggestions for research needed to further develop AI tools that are safe, culturally appropriate and relevant to crisis-affected populations. It calls for stronger evaluation and evidence, attention to clinical safety and practice, consideration for equity and access and governance models that prioritise local ownership and accountability. The relevance of this work is that it can inform global policy discussions on digital health in emergencies, while also offering concrete guidance for the research needed. Ultimately, this article supports a shift from rapid, uncoordinated adoption of AI towards approaches that protect human relationships, strengthen frontline care and ensure that technological advances benefit those most affected by humanitarian crises.

## Introduction

In 2025, armed conflict, climate disasters and mass displacement left 305 million people worldwide in need of assistance (UN, 2025). Crisis-affected populations have an increased burden of mental, neurological and substance-use disorders relative to non-affected populations, with prevalence estimates for common conditions such as depression, anxiety and post-traumatic stress disorder (PTSD) two- to threefold higher than those in stable settings (Blackmore et al., 2020; Carpiniello, 2023). The resulting psychological burden far outstrips existing local capacities, leaving 75–85% of those with severe disorders in low- and middle-income countries without any access to care (InterAction, 2022; WHO, 2025b). This treatment gap is exacerbated in humanitarian crises by a combination of factors, including limited and poor coordination of appropriate services, variability in quality of care, unstable funding for mental health, restricted local workforce capacity, stigma and fear of discrimination and preferences for self-management or informal help (Echeverri et al., 2018; Tol et al., 2020; Troup et al., 2021). Given rising humanitarian needs alongside significant funding shortfalls, this gap is likely to widen further in the coming years (Global Mental Health Action Network, 2025). Against this backdrop, artificial intelligence (AI) is increasingly proposed to scale mental health support (Cruz-Gonzalez et al., 2025; Humayun et al., 2025). Recent systematic reviews report that AI-based conversational agents can produce short-term reductions in depression and anxiety symptoms, with moderate-to-large effect sizes, and that AI methods can support diagnostic classification, treatment monitoring and clinician-facing tasks (Cruz-Gonzalez et al., 2025; Humayun et al., 2025). However, this evidence is predominantly from high-income countries and non-crisis populations; both reviews note the absence of evaluation in low-resource and crisis-affected settings. Whether AI can deliver comparable benefits in humanitarian contexts, and what risks it introduces there, remains an open question.

In the context of humanitarian crisis response, there are distinct challenges to the use of AI tools, including fragmented data systems, high population mobility, linguistic and cultural diversity and weak digital infrastructure (Guha-Sapir and Scales, 2020; Colombo and Altare, 2025). Recent research by the Humanitarian Leadership Academy and Data Friendly Space has reported a “Humanitarian AI Paradox”: while 70% of aid workers report using AI weekly, and 69% of staff report reliance on commercial platforms (e.g., ChatGPT and Claude), fewer than 25% of organisations had established a formal AI strategy (Johnson et al., 2025). What makes the need for attention to this paradox specifically acute in humanitarian mental health and psychosocial support (MHPSS) is the combination of three features: (1) the affected population is, by definition, in acute distress and exposed to heightened power and dependency dynamics in relation to those providing assistance. This limits their ability to refuse, contest or meaningfully consent to how AI tools mediate their care (Kreutzer et al., 2025), which is heightened when the care in question involves disclosure of trauma, suicidality or other information with protection implications; (2) clinical interpretation depends on culturally and linguistically shaped expressions of distress (Ventevogel et al., 2013), where misreading an idiom can lead to wrongful escalation, inappropriate diagnosis or missed risk, which is difficult to reverse; and (3) care is delivered across a wide treatment gap by a largely non-specialist workforce (Saxena et al., 2007; Connolly et al., 2021), who may rely on AI-generated guidance for high-stakes decisions without being well placed to recognise when it is wrong.

The existing governance vacuum, whereby AI use outpaces institutional safeguards, creates risks for crisis response, particularly in the context of providing care for trauma-affected populations. These risks are compounded by the inherent technical limitations of the tools; current automated systems reportedly struggle to convey empathy or context-sensitive judgement (Higgins et al., 2023; Montag et al., 2024), which are key in MHPSS crisis response (Tay et al., 2019; Goodwin and Kraft, 2022). As such, the gap between informal AI adoption and institutional readiness is not a temporary lag but a structural risk that requires sector-specific attention to which AI capabilities are emerging, where they can be safely applied, and what safeguards are needed for tools that are already in widespread use.

Recent global research and policy developments reflect this growing recognition. A common reservation about AI-based MHPSS in crisis-affected settings is that intended users lack the connectivity to access such tools; however, this objection is increasingly out of date. Research by UNHCR has shown that refugee populations prioritise connectivity as a survival tool, with a report published in 2016 indicating that 93% of all refugees live in places covered by at least a 2G network (UNHCR, 2016). Subsequent global digital indicators, such as the World Bank ICT data (World Bank, 2026), show continued and rapid growth in mobile broadband coverage and smartphone access in low- and middle-income countries, including in many crisis-affected regions. Limited connectivity remains a real equity issue for specific groups, but it is no longer a credible reason to defer investment in AI-supported MHPSS at the sector level.

In terms of policy, the 2024 World Health Assembly endorsed a resolution to strengthen MHPSS at all stages of emergency response, noting that safe digital technologies may support these aims (WHO, 2024). While the World Mental Health Report (WHO, 2022) and the humanitarian MHPSS research agenda 2021–2030 (Tol et al., 2023) identify digital approaches as a priority, they do not explicitly address generative AI, data governance or safe deployment in crisis contexts. Since these publications, rapid advances have taken place in the field of AI. This article aims to discuss key opportunities, challenges and research needs for using AI in humanitarian MHPSS response. We will first review the current advances in AI and the opportunities relevant to MHPSS response, which differ fundamentally from previous digital health tools in their autonomy, scalability and economic accessibility.

Throughout, we use AI to refer to generative AI systems built on large language models (LLMs), which are neural networks trained on large bodies of text (and increasingly other modalities) that produce text-based responses to natural-language prompts. Where relevant, we also discuss small language models (SLMs), which are smaller, less computationally demanding LLMs that can be run on local devices, and retrieval-augmented generation (RAG), an architecture in which an LLM’s outputs are grounded in a specific reference library (e.g., clinical guidelines) retrieved during response.

## AI developments relevant to humanitarian MHPSS response

We consider that four concurrent trends are currently reshaping the technical feasibility, economic viability and strategic opportunity of AI deployment within MHPSS response in the humanitarian sector.

### First, the rise of agentic AI

Agentic AI refers to systems designed to act as autonomous agents that can plan, make decisions and carry out sequences of actions to reach a goal. Unlike traditional AI that responds to a prompt or performs a narrow task, agentic systems can decide what to do next, use tools and adapt their behaviour based on results. From a humanitarian perspective, through the so-called RAG, these agents can ground their outputs within key guidelines, such as the mhGAP Humanitarian Intervention Guide (WHO and UNHCR, 2015), and the Sphere Handbook, which are the global minimum standards for humanitarian response (Sphere Association, 2018). For example, such systems could support community health workers by grounding guidance in mhGAP protocols during supervision or triage. While these systems can use internal self-evaluation to flag uncertainty for human review, their autonomous nature requires robust accountability frameworks, mechanisms that are currently absent from humanitarian AI governance. In mental healthcare, the consequences of weak accountability are particularly serious: if an AI misreads a local expression of grief as symptoms of psychosis and a clinician accepts this label without challenge, the result can be wrongful escalation, inappropriate medication or unnecessary hospitalisation. This tendency to accept AI-generated information without critical reflection, known as automation bias, has been shown to lead clinicians to follow incorrect AI recommendations at substantially higher rates (26%) than they would otherwise (Goddard et al., 2012). These effects are amplified under high workload, time pressure and limited experience, conditions characteristic of humanitarian frontline work (Goddard et al., 2012).

### Second, the emergence of an agentic workforce

AI agents are increasingly operating as team members alongside human workers, and many technology companies are actively pushing for this. In MHPSS, the appeal is strongest where treatment gaps are wide and documentation burdens are high. AI agents can be used to streamline case notes, produce structured supervision summaries, support translation and adaptation of materials, and suggest guideline-aligned options. However, for humanitarian MHPSS response, it can import assumptions about productivity into settings where relationship and rapport building are often a key part of the intervention. What counts as an efficiency gain in corporate workflows may be a clinical loss in MHPSS if it reduces active listening, presence or trust building. This is particularly risky for trauma-affected populations and communities who have justified concerns about surveillance or misuse of sensitive information. The central governance question is therefore not whether AI agents work, but which tasks can and should be delegated without weakening care.

### Third, open-source AI models

Open-weight models, those whose underlying parameters are publicly released, allowing anyone to download, run and modify them (i.e., DeepSeek R1, Qwen 3, Llama and Mistral), are performing equally well or better than leading proprietary models, which are accessed only through the developer’s servers (i.e., GPT-4o) (Sandmann et al., 2025; Stachura et al., 2025). In practice, the availability of open-source models means they can be run locally, fine-tuned on new data and used in secure environments. For humanitarian MHPSS, this matters because, in theory, it makes high-quality models usable under tighter control: organisations can fine-tune on region-specific protocols, run systems offline in low connectivity settings and adopt community-led governance without sending sensitive data to external corporate APIs, for example. This is potentially relevant for MHPSS serving displaced populations where data sensitivity is essential, and offline use could be transformative, though, to our knowledge, empirical evaluations of fine-tuned open-source AI models in humanitarian MHPSS settings are not yet available.

However, open-weight also relocates responsibility. With proprietary models, the developer retains control over safety testing, content moderation and clinical guardrails. With open-weight models, if a humanitarian organisation downloads and adapts a model, that duty sits primarily with the deployer (i.e., an NGO) rather than the vendor (i.e., DeepSeek R1). In a sector already marked by limited AI capacity and governance, there is a real risk of open-weight models being rolled out without sufficient clinical containment, increasing the chance of unsafe guidance or discriminatory outputs, with direct implications for triage, risk assessment and frontline decision-making, among others.

### Fourth, the collapse of inference costs

The rapid decline in AI inference costs may affect the feasibility of digital tools in humanitarian health. Inference is the cost of running a trained model in real time, such as answering questions or translating text. Recent analyses suggest that the price to achieve a given level of AI performance has been declining roughly tenfold per year (Gundlach et al., 2025). Whether this trajectory continues, plateaus or reverses is uncertain and depends on hardware, regulation and energy costs. If even a fraction of these gains continues, applications that were prohibitively expensive 2 years ago, like real-time translation or quality monitoring, will become more economically feasible. Lower costs could, in turn, make multilingual psychoeducation, supervision support and translation of clinical encounters, for example, more feasible in resource-constrained settings. Though feasibility will depend on language coverage, safety testing and integration costs that are not captured in the per-token price.

However, these gains are not evenly shared across languages. Because models are optimised for English-language performance, non-English languages systematically require more tokens to express the same meaning. For example, a quick analysis of the token tariff on different EU languages showed an ~87% higher token use for Dutch, 151% for Polish and 94% for Portuguese (van der Boor, 2025). Since AI providers typically bill organisations per token processed, this technical inefficiency translates directly into a financial tax, creating a built-in cost and performance penalty for non-English interactions. The performance gap compounds the cost gap: in a recent psychiatric evaluation, an open-source LLM produced significantly more hallucinations and significantly lower diagnostic accuracy when processing clinical notes in Korean compared to their English translations (Kim et al., 2025). In humanitarian MHPSS, where many languages differ further from English, the same budget buys fewer and less safe interactions for the people most in need. Some efforts, such as language-specific tokenizers and targeted training data, reduce this gap, but they tend to follow market demand, leaving lower-resource languages less supported and more costly. Addressing this gap will likely require coordinated investment by multilateral agencies, humanitarian donors and research partnerships to develop tokenizers and training datasets for underrepresented languages.

These technological developments do not determine how AI should be used in humanitarian mental health care, but they shape which applications are technically and economically feasible. Their implications depend on how systems are governed, which tasks are delegated to AI and how risks related to safety, bias, and accountability are managed within fragile service environments and rapid response. They also raise questions of equity, as many crisis-affected populations rely on languages that remain poorly represented in current models.

We use the multi-layered intervention pyramid from the 2007 Inter-Agency Standing Committee Guidelines on MHPSS (IASC, 2007) as an organising framework to show how AI capabilities might apply to MHPSS response in humanitarian contexts. The IASC Guidelines were developed through inter-agency consensus and are the most widely endorsed framework for organising MHPSS in humanitarian response, structuring services into four progressively specialised layers. The following section examines how the emerging AI capabilities might translate into concrete applications across the levels of the IASC MHPSS pyramid.

## Opportunities and risks for AI in MHPSS response

Table 1 provides illustrative examples of how recent AI capabilities might apply at each of the four levels of the IASC pyramid (IASC, 2007): (1) basic services and security, (2) community and family support, (3) focused non-specialised support delivered by trained workers and (4) specialised services delivered by mental health professionals. To distinguish what is usable now from what requires further development, the table separates *current applications*, that is, those feasible now with appropriate safeguards, from *emerging applications*, which are technically feasible but require further benchmarking and evaluation before responsible use in humanitarian settings. Direct evaluations of these applications in humanitarian MHPSS contexts remain a key research gap. Any use should align with established humanitarian and health governance frameworks, including the IASC Guidelines on MHPSS in Emergency Settings (IASC, 2007), Sphere Standards handbook (Sphere Association, 2018) and WHO guidance on ethics and governance of AI for health (WHO, 2025a).

**Table 1.** Examples of how recent AI capabilities (agentic workflows, co-pilots, open-weight models and lower inference costs) might apply across the IASC MHPSS pyramid Table 1. long description.

AI applications (selected examples)		
IASC pyramid level	Current applications (feasible now with appropriate safeguards)	Emerging applications (technically feasible, requiring development of benchmarks and evaluation)
**Level 1: Basic services and security** Ensuring basic services and security are provided in socially and psychologically supportive ways	Auto-generated situation reports synthesising fragmented field data Multilingual safety messaging for displaced populations Document processing and digitisation of paper-based intake forms AI-assisted matching of displaced families with available services (shelter, health, family tracing and other) Satellite imagery analysis combined with LLMs for rapid needs assessment in newly accessible areas	Natural language processing analysis of helpline transcripts and social media to detect emerging distress patterns at population level Machine-learning forecasts of displacement routes during floods or conflict to pre-position WASH, shelter and protection services
**Level 2: Community and family support** Focused on community-level support	Translation of psychoeducation and self-care materials into local languages AI-assisted adaptation of materials to local literacy levels Chatbot-supported information dissemination in displacement settings AI-assisted training material development for community volunteers and lay helpers	Conversational agents grounded in validated MHPSS guidelines and protocols could deliver psychoeducation and self-care guidance in local languages and culturally adapted Natural language processing analysis of social media, community radio transcripts or messaging-app feedback groups could help detect emerging concerns in the social environment (e.g., fears of attacks, stigma or discrimination) Voice-based interfaces for low-literacy populations to access self-care content AI-supported moderation of peer-support platforms (flagging concerning content for human review)
**Level 3: Focused, non-specialised support** Focused on people who require focused individual, family or group interventions by trained and supervised workers.	AI-assisted generation of structured clinical notes and case summaries Real-time translation of clinical encounters Summarising case logs or highlighting cases that may require review (i.e., triaging)	LLMs may be most immediately useful at Level 3 because supervision, training, documentation and protocol adherence are bottlenecks LLM-based tutors could potentially simulate client interactions for community health worker training (role-play with feedback) Real-time prompts to community health workers during sessions based on protocol Automated competency assessment for community health worker training and certification Decision-support systems could be designed to ground responses in mhGAP-HIG protocols rather than generic training data
**Level 4: Specialised services** Support for the small percentage of people requiring specialised care	Drafting of clinical case summaries for telepsychiatry consultations Co-pilot for psychiatrists managing high caseloads remotely	Potential agentic prioritisation of high-severity patients for limited specialist time AI-assisted treatment planning grounded in clinical guidelines
**Across levels**	Speech-to-speech translation across languages Structuring and cleaning data Document processing AI-assisted clinical letter writing for referrals between non-specialists and specialists AI-assisted literature search and evidence synthesis for the latest available evidence, unfamiliar clinical presentations or rapidly evolving guidance	Agentic AI co-pilots for end-to-end documentation workflows Pre-deployment crisis simulations Synthetic data generation for rare clinical training scenarios

*Note:* These are illustrative rather than exhaustive and are not based on empirical evaluations in humanitarian MHPSS contexts, which to our knowledge are not yet available. The distinction between current and emerging applications reflects readiness for responsible use rather than technological novelty: current applications are feasible now with appropriate safeguards, while emerging applications require further benchmarking and evaluation before use in humanitarian settings. The cells in this table reflect technology that already exists; however, the benchmarks and regulatory frameworks do not exist yet particularly for the emerging applications. For practitioners or policymakers to make ethical and responsible recommendations, empirical evidence is on effectiveness and implementation across humanitarian contexts is needed.

These examples show opportunities for where AI could support MHPSS, yet translating technical capability into safe practice is not straightforward. MHPSS involves the interpretation of culturally shaped expressions of distress, the management of acute emotional states and decisions with safeguarding implications. These challenges are intensified in humanitarian settings characterised by displacement, which has been described by Bhatnagar et al. (2025), including limited organisational readiness, low-quality or fragmented data, unstable infrastructure, power imbalances between local and external actors, surveillance risks and sustainability concerns. MHPSS applications introduce several additional risks that are specific to mental health:

### Misreading cultural expressions of distress

Mental health conditions often manifest through culturally shaped idioms of distress, somatic complaints, spiritual explanations and language and symptoms that vary across populations (Cork et al., 2019, Kirmayer et al., 2017). AI systems trained predominantly on Western clinical taxonomies may misinterpret these expressions, leading to inappropriate or absent referrals (Kim et al., 2025). This is not inherent to AI; human providers can also struggle with cultural interpretation, but AI systems lack the relational context that allows clinicians to probe, clarify and adapt. These risks are compounded in non-English languages, where model performance is further degraded, as discussed above.

### Over- or under-pathologising crisis reactions

A related risk is the misclassification of normal coping reactions to abnormal circumstances as clinical disorders, or conversely, failing to identify genuine suffering expressed through somatic language, metaphor or behavioural withdrawal. AI screening tools calibrated to clinical populations may not distinguish adaptive distress from pathology in crisis contexts. Generative systems may also produce confident, persuasive explanations for incorrect classifications and their outputs can vary across near-identical prompts, making errors harder to anticipate, reproduce and audit in clinical workflows. Avoiding this requires both domain expertise and training in how to use AI appropriate for such workflows.

### Amplifying bias

Several biases that exist in general AI use are likely to be further increased in crisis contexts, which has an impact on MHPSS response. First, as previously described, automation bias is well-documented and may increase under time pressure, uncertainty and among non-specialist workers (Goddard et al., 2012; Lyell and Coiera, 2017). Second, language models show a tendency to align responses with users’ expressed beliefs over truthful ones, a behaviour known as sycophancy (Sharma et al., 2024). In mental health interactions, this carries particular risk: a model that echoes a user’s negative thoughts rather than gently challenging them could deepen distress rather than ease it. These risks are not unique to humanitarian MHPSS, but they are likely to be magnified in populations with elevated rates of trauma, depression and anxiety, and in workforces with potentially limited expertise and capacity to second-guess confident-sounding AI outputs.

### Lack of containment and risk management

General-purpose AI systems today are not trained to apply specific therapeutic techniques, such as grounding, and will struggle to pick up cues to respond to dissociation or provide the interpersonal safety required during moments of acute distress. Without capacity for containment, there is a risk of (re)traumatisation, overwhelm or abandonment. Humanitarian workers are trained to assess self-harm risk, recognise safeguarding concerns and initiate protective referrals. Current AI systems cannot reliably perform these tasks. This unpredictability is particularly problematic in safeguarding situations, where consistent responses and traceable decision logic are needed.

### Data governance and consent

This is particularly acute for refugee and displaced populations. If data governance is weak, information about mental health status, trauma disclosures and/or psychosocial distress could be accessed by third parties, exposing individuals to physical and/or social risks. When LLMs are connected to case management systems or automated workflows that can perform actions, additional measures, such as limiting access to authenticated users, should be taken to avoid potential data exfiltration through LLM-specific risks like prompt injection attacks. Prompt injection attacks are where manipulated inputs are used to override system instructions or trick the model into revealing sensitive information or performing unauthorised actions.

These risks are compounded by the difficulty of getting genuine informed consent in humanitarian settings. Crisis-affected populations often depend on services with limited alternatives, and refusing data collection could be seen as risking access to assistance (Kreutzer et al., 2025). AI adds further challenges: lay people may not understand AI processing, future uses of data by commercial models cannot be fully specified, and data used to train or fine-tune models currently cannot be withdrawn (Kreutzer et al., 2025). In this context, meaningful consent may be difficult to guarantee, and mental health information may carry particular risks for individuals, as disclosures can lead to stigma and family, legal, social or protection-related harms.

### Disruption of trust and community-based coping

If AI reduces human connection in MHPSS, it risks undermining trust in communities with histories of trauma, state violence and institutional mistrust, potentially increasing withdrawal and reducing help-seeking. Trust also operates on the provider side: if tools are poorly integrated, unclear or misaligned with workflows, clinicians may either avoid them altogether or adopt informal workarounds, potentially creating new safety and privacy risks. AI tools may also focus primarily on individualised screening and referral, neglecting collective forms of resilience, peer support and traditional healing networks that are central to recovery in many cultural contexts.

### The benchmarking gap

In other domains, validated benchmarks are being developed to evaluate AI performance (e.g., SWE-Bench for software engineering [Rashid et al., 2025] and MedQA for clinical knowledge recall [Jin et al., 2021]). Most of these are not conversational, and those that are, like Healthbench by OpenAI (Arora et al., 2025), are 85% in English and do not focus on mental health. No equivalent frameworks exist for AI in humanitarian MHPSS. Without rigorous evaluation, limitations may only be discovered through error and potential harm. Developing context-specific benchmarks, that is, co-designed with local mental health professionals, affected communities and humanitarian practitioners, should be a research priority.

### Who builds these systems?

Foundation models are developed by technology companies (e.g., OpenAI, Anthropic, Google DeepMind and DeepSeek) with commercial priorities. This raises questions of technological dependency, vendor lock-in and reliance on tools poorly suited for applications in humanitarian MHPSS, as they were designed for other purposes.

### Synthetic data: Opportunity and risk

AI-generated synthetic data could increase sparse training datasets for humanitarian contexts. However, as data generation becomes easier, creating methods for ensuring data quality becomes more important. New approaches to track data provenance, verify authenticity and ensure quality are needed to ensure high-quality data can be used to train and evaluate AI applications in context.

While the risks are presented as a list, they do not all operate at the same level of the IASC pyramid. Table 2 summarises each risk and indicates the levels at which we consider it to be most acute.

**Table 2.** Summary of risks in the use of AI for humanitarian MHPSS, mapped to IASC pyramid levels Table 2. long description.

Risk	Summary	IASC levels most affected
Misreading cultural expressions of distress	AI trained on Western taxonomies misinterprets local culture and idioms of distress	All levels (especially 2, 3, 4)
Over- or under-pathologising crisis reactions	Misclassifying adaptive distress as pathology, or missing genuine suffering	Levels 3, 4
Amplifying bias	Automation bias, sycophancy and confirmation bias magnified in distressed populations	All levels (especially 2, 3)
Lack of containment and risk management	AI cannot reliably manage acute distress, dissociation or safeguarding	Levels 3, 4
Data governance and consent	Sensitive disclosures, weak consent, lack of transparent data flows and ownership	All levels
Disruption of trust and community-based coping	AI reducing human connection, collective resilience and imposing an individualistic approach to support	Levels 1, 2, 3
The benchmarking gap	No validated evaluation frameworks for humanitarian MHPSS	All levels
Who builds these systems	Commercial priorities, vendor lock-in and technological dependency	All levels
Synthetic data: opportunity and risk	Opportunities for scarce data offset by quality and provenance risks	All levels

## Research needs

In response to the identified opportunities and risks, we propose the following research needs to develop evidence that can guide safe practice and equitable use of AI for MHPSS response in emergencies, organised across four areas:

1. Evaluation and evidence – *What we need to know:*
  - **Standardised benchmarking:** What evaluation frameworks and datasets are needed to assess AI performance in humanitarian MHPSS for the various use cases in each IASC tier, and how can these be co-developed with affected communities to ensure clinical and ethical relevance? How to make these widely available for anyone to be able to evaluate their AI applications before using them in practice, but also to contribute “ground-truth” data?
  - **Effectiveness and cost-effectiveness across IASC levels:** Which AI-enabled use cases improve outcomes, quality, access or equity at each IASC tier compared with non-AI alternatives, and what are the costs and associated risks?
  - **Coordinated agenda-setting:** How can key global, national and local actors, including humanitarian coordination bodies, MHPSS technical working groups, affected communities, researchers and funders, be convened to establish consensus on research priorities, evaluation standards and shared infrastructure for AI in humanitarian MHPSS?
  - **Co-design of evaluation criteria:** What methods enable communities to define success criteria for AI tools before development begins? How can evaluation frameworks balance community-defined outcomes (e.g., respect for traditional healing and maintenance of trust) with clinical efficacy measures?

1. Clinical safety and practice – *How AI interacts with care:*
  - **Clinical safety and containment:** What safeguards are needed when AI tools engage with individuals in acute distress? How should handoff to human providers be designed, including hybrid forms of working?
  - **Workforce transformation:** Beyond simple “task-shifting,” how does AI integration impact provider practice (e.g., automation bias, deskilling or informal workarounds), and what supervision models best mitigate these risks?
  - **The clinical encounter:** How does the introduction of AI alter the care-seeking trajectory for patients, and “therapeutic alliance” particularly for those who may lack alternative options?
  - **Cultural calibration:** How can AI systems be designed to recognise culturally specific idioms of distress, and how does AI’s interpretation of these idioms differ from, or reinforce, human clinical bias? What governance and ownership structures ensure that documentation of local idioms remains under community control, and that knowledge extraction does not reproduce colonial patterns of data extraction?

1. Equity and access – *Who benefits and who is left out:*
  - **Linguistic equity:** How do LLM performance and cost vary across languages spoken by crisis-affected populations? What approaches can reduce the multilingual processing penalty?
  - **Technological resilience:** In settings with zero connectivity or unstable power, can “Small Language Models” (SLMs) be provided to run on limited power edge devices while maintaining the clinical nuance and safety required for MHPSS?
  - **Environmental sustainability and resource scarcity:** How do the global energy and water demands of AI infrastructure interact with humanitarian principles, given that the climate consequences of these demands may fall disproportionately on the low- and middle-income countries where many crisis-affected populations live? What governance approaches can ensure that AI adoption in humanitarian MHPSS is consistent with environmental commitments at the sector and donor level?
  - **Digital inclusion:** How can AI-supported MHPSS be made accessible to persons with physical, sensory or cognitive disabilities, and what are the specific risks of their exclusion from standard algorithmic workflows?
  - **Intersectional equity in digital access:** How does digital connectivity in crisis-affected populations differ by gender, age, literacy, ethnicity and socioeconomic conditions, and how do these dimensions interact? Given that those least likely to have stable digital access often overlap with those at the highest MHPSS need, what design and delivery approaches can prevent AI-supported services from systematically widening rather than closing the access gap?

1. Governance and accountability *– Who decides and who is responsible:*
  - **Agentic AI governance:** As systems move towards autonomous, multi-step tasks, what frameworks are needed to validate agentic workflows and maintain human accountability for clinical outcomes?
  - **Governance models and political economy:** What accountability structures can prevent technological dependency on commercial entities, and how can research ensure that AI models and the insights generated from crisis settings are owned by the communities they intend to serve?

Addressing these research needs is not only a technical challenge but also a question of power, representation and epistemic justice. AI systems trained predominantly on Global North datasets may standardise and homogenise culturally diverse and fluid expressions of distress, privileging forms of suffering that align with dominant psychiatric categories while marginalising others. Poorly governed systems, therefore, risk contributing to epistemic injustice. Addressing these challenges requires coordinated action grounded in social justice and decolonial approaches, including the development of locally grounded datasets, meaningful participation of affected communities and governance frameworks that recognise diverse knowledge systems and cultural understandings of distress.

## Coordinated action and commitment to key principles

Supporting the safe, equitable and culturally grounded use of AI in humanitarian MHPSS requires coordinated action grounded in principles of social justice, decolonisation, epistemic justice and community ownership.

Local governments, affected communities and survivor and community networks need to be adequately resourced and positioned to lead governance and monitoring of AI systems. This includes the right to determine whether and how AI is used in their care, not only to oversee tools built by others. Particular attention is needed for groups facing distinct data exposure and consent risks, including but not limited to children, survivors of gender-based violence and persons with disabilities, among others.

Academic institutions need to prioritise epistemic justice: a commitment to centring local knowledge systems, lived experience and culturally grounded understandings of mental health as the “gold standard” for evaluating AI, rather than relying solely on Global North psychiatric benchmarks. In practice, this means co-developing evaluation criteria with communities before tools are built, not validating tools against community feedback after they have already been developed. Humanitarian coordination bodies (e.g., IASC and MHPSS Technical Working Groups) should establish red lines and safety protocols that protect the human relationship, ensuring that data and the logic of these tools remain under the ownership of the communities they intend to serve.

AI developers need to move beyond extractive or consulting approaches towards models grounded in technological sovereignty, meaning communities control how their data is collected, owned and used and equitable partnerships. This includes ensuring that local clinicians, researchers, AI developers and affected communities have the authority and resources to shape AI models, tuning it to, for example, recognise local idioms of distress and ensuring it can function in the low-connectivity environments where care often happens. Success in this model is already visible in initiatives like Jacaranda Health’s PROMPTS in Kenya (https://jacarandahealth.org/prompts/), which combines AI-supported triage with human-led maternal care, or the Butabika National Referral Hospital in Uganda, where AI is locally led to support mental health call centres (https://www.butabikahospital.go.ug/news/mental-health-innovation-with-ai). When developed through participatory and locally led approaches, AI systems should create opportunities for communities to generate culturally grounded psychoeducational materials, document local idioms of distress and shape digital mental health tools, among others, according to their own conceptualisations of well-being and recovery.

Funders (e.g., ECHO, FCDO and Wellcome Trust) should support long-term and locally led research rather than short-cycle pilot projects. Funding mechanisms should require independent evaluation, participatory harm monitoring, transparency and the publication of null or negative findings to reduce bias and prevent overstated claims of effectiveness. Given the elevated and specific mental health needs that occur during and after crises, dedicated investment is needed to support the safe, culturally grounded and equitable use of AI in humanitarian settings.

## Conclusion

AI is likely to reshape humanitarian mental health response, yet its value lies not in the replacement of human providers, but in the strategic support of communities and the frontline workforce. We see strong potential at Level 3 of the IASC pyramid, where AI-driven decision support and documentation tools can alleviate bottlenecks in supervision and training, allowing providers to focus their limited capacity on empathy and context-sensitive judgement. However, the success of this integration depends on getting the human-AI relationship right within fragile service environments and on closing the linguistic, cultural and evaluation gaps that currently disadvantage those most in need. Turning this potential into safe practice requires locally led evidence-based research and governance models that centre community ownership and epistemic justice.

The Humanitarian AI Paradox, where informal AI use is already outpacing institutional safeguards, means the sector cannot afford to wait. Standards, benchmarks and guardrails developed now should ensure that these tools strengthen rather than compromise the well-being, rights and care of populations affected by crisis.

## Long descriptions

### Table 1. Long description

The table is organized into three columns: I A S C pyramid level, Current applications (feasible now with safeguards), and Emerging applications (requiring benchmarks and evaluation).

* Level 1: Basic services and security. Current uses include auto-generated situation reports, multilingual safety messaging, and satellite imagery analysis with L L Ms. Emerging uses include N L P analysis of helpline transcripts and machine-learning forecasts for displacement routes.

* Level 2: Community and family support. Current uses include translation of psychoeducation materials and chatbot-supported information. Emerging uses include conversational agents for self-care guidance and voice-based interfaces for low-literacy populations.

* Level 3: Focused, non-specialized support. Current uses include A I-assisted clinical notes and real-time translation of encounters. Emerging uses include L L M-based tutors for health worker training and decision-support systems grounded in m h G A P-H I G protocols.

* Level 4: Specialized services. Current uses include drafting case summaries for telepsychiatry and co-pilots for psychiatrists. Emerging uses include agentic prioritization of high-severity patients and A I-assisted treatment planning.

* Across levels: Current uses include speech-to-speech translation and evidence synthesis. Emerging uses include agentic A I co-pilots for documentation and synthetic data generation for training.

Navigate back to Table 1..

### Table 2. Long description

The table consists of three columns: Risk, Summary, and I A S C levels most affected.

* Misreading cultural expressions of distress: A I trained on Western taxonomies misinterprets local culture and idioms of distress. Affects all levels, especially 2, 3, 4.

* Over- or under-pathologising crisis reactions: Misclassifying adaptive distress as pathology, or missing genuine suffering. Affects levels 3, 4.

* Amplifying bias: Automation bias, sycophancy and confirmation bias magnified in distressed populations. Affects all levels, especially 2, 3.

* Lack of containment and risk management: A I cannot reliably manage acute distress, dissociation or safeguarding. Affects levels 3, 4.

* Data governance and consent: Sensitive disclosures, weak consent, lack of transparent data flows and ownership. Affects all levels.

* Disruption of trust and community-based coping: A I reducing human connection, collective resilience and imposing an individualistic approach to support. Affects levels 1, 2, 3.

* The benchmarking gap: No validated evaluation frameworks for humanitarian M H P S S. Affects all levels.

* Who builds these systems: Commercial priorities, vendor lock-in and technological dependency. Affects all levels.

* Synthetic data: opportunity and risk: Opportunities for scarce data offset by quality and provenance risks. Affects all levels.

Navigate back to Table 2..
