# Supplementary material for: The humanitarian AI paradox: Key opportunities, challenges and research needs for the use of AI in humanitarian mental health response
Source: Glob Ment Health (Camb). 2026 Jun 16;13:e130. doi: 10.1017/gmh.2026.10254 (PMC13373262; doi:10.1017/gmh.2026.10254)
Supplement: Peer review history

# Reviewer report: Review: The humanitarian AI paradox: Key opportunities, challenges and research needs for the use of AI in humanitarian mental health response — R0/PR2

**DOI:** 10.1017/gmh.2026.10254.pr2

This manuscript addresses an important and timely topic. The focus on artificial intelligence in humanitarian mental health and psychosocial support is highly relevant, and the paper has clear potential to make a useful contribution. The idea of the “Humanitarian AI Paradox” is interesting, and the manuscript raises several important concerns around governance, safety, culture, language, and accountability.

However, in its current form, the paper still requires major revision before it is ready for publication. First, the main argument needs to be sharper and more focused. The concept of the “Humanitarian AI Paradox” is promising, but it is still defined too broadly. The paper would be stronger if the authors explain more clearly what is unique about this paradox in humanitarian mental health settings, and how it goes beyond general concerns about rapid AI adoption and weak governance.

Second, the paper would benefit from a tighter structure. Some sections read more like a broad overview than a focused perspective argument. In particular, the discussion of AI developments, risks, and future opportunities should be linked more directly to the central purpose of the paper. At present, there are many good points, but the overall argument sometimes feels too wide and not sufficiently prioritised.

Third, several claims need more careful framing. Some statements about model performance, falling costs, open-weight models, and future feasibility are expressed too confidently. Since this is a perspective paper, it is acceptable to discuss emerging trends, but the authors should distinguish more clearly between evidence-based conclusions and forward-looking expectations.

Fourth, the research agenda is relevant and valuable, but it is currently too long and reads more like a list of all possible future questions. The paper would be more useful if the agenda were prioritised into a smaller number of core areas, such as safety, cultural and linguistic validity, governance, and effectiveness in practice.

Fifth, the paper should improve the use of examples and supporting references. Some examples are helpful, but they are not fully integrated into the main argument. In addition, a few references are weaker than expected for key claims. Stronger support should be provided where possible, especially for factual or comparative statements.

Sixth, Table 1 is useful, but it would be stronger if it separated near-term realistic applications from more advanced or speculative possibilities. At the moment, some examples feel practical and immediate, while others feel more aspirational, and these should be distinguished more clearly.

Finally, the paper would benefit from a careful language and style edit. The manuscript is readable overall, but some sentences are too long, repetitive, or awkwardly phrased. A cleaner writing style would improve clarity and strengthen the paper’s impact.

Overall, this is a thoughtful and relevant paper with real potential, but it needs clearer framing, stronger prioritisation, and a more disciplined presentation before it can be considered for publication.

# Reviewer report: Review: The humanitarian AI paradox: Key opportunities, challenges and research needs for the use of AI in humanitarian mental health response — R0/PR3

**DOI:** 10.1017/gmh.2026.10254.pr3

This is a valuable paper providing much needed technical, analytical and practical guidance on the topic of AI for mental health in humanitarian crises. The following are some suggestions to try and further improve the paper.

Title:

The title focuses on a research agenda but I don’t think the paper delivers on that (see below). Instead I think it provides a very helpful analysis of key opportunities and challenges and research needs. I suggest it be re-phrased accordingly.

Abstract:

The abstract will need updating if the points below are addressed.

The abstract should note that such settings represent ‘cultural and linguistic diversity’ rather than just ‘linguistic diversity’.

Background:

It would be good to note the elevated burden of mental health disorders among crisis-affected populations.

It would be good to include a very brief one-sentence description of the reasons why AI is seen as beneficial for MHPSS in humanitarian contexts. i.e. the rational for focusing on AI in this paper could be strengthened (i.e. after the sentence ‘Against this

backdrop, artificial intelligence (AI) is increasingly proposed to scale mental health support’). I appreciate this is addressed further down in the paper but the reader needs some brief explanation on the potential value of AI in order to understand the importance of the issue/paper.

The paper could make a stronger point about rising data coverage among crisis-affected populations/LMICS, in order to pre-empt criticism on limited relevance of AI-related services due to limited data coverage. This should include greater use of sources (e.g. https://datareportal.com and World Bank data). Research funders/reviewers remain stuck in this argument as a reason not to invest in AI for MHPSS and so it’s helpful to push back against such an argument.

Aim:

The aim does not necessarily match the structure of the paper. Having read the aim, the reader expects to first read about opportunities but is instead presented with a description of ‘AI Developments Relevant to Humanitarian MHPSS response’. This is very helpful but not reflected in the aim. It could therefore be good to revise the aim to something like: ‘To discuss developments, opportunities, risks and research needs for using AI in humanitarian mental health response’.

It is also a bit odd to include a means of analysis (IASC) and reference in an aim. Therefore, it would be better to mention the IASC framework when presented later on in the paper (i.e. in the ‘Opportunities and Risks for AI in MHPSS Response’ section). This will also improve the flow of the aim. In addition, many readers will not be familiar with the IASC guidelines and so a brief description should be given and a rationale for their use (i.e. their legitimacy) when they are first mentioned in this section in the main paper.

AI Developments Relevant to Humanitarian MHPSS response:

This is a very helpful and valuable section.

It is noted that ‘we consider six concurrent trends are current…’. As far as I can see, only four trends are mentioned (agentic AI, agentic workforce, open-source AI models, collapse of inference costs)?

Opportunities and Risks for AI in MHPSS Response:

See above point about describing IASC guidelines here and rationale for their use.

In addition, readers may not be familiar with Sphere and so a very brief explanation is also required for them.

Paragraph two in this section refers to ‘domain specific risks’. It’s not clear how the eight points presented are domains. What does a domain mean in the context of these risks?

I’m not sure having a detailed table of opportunities and then text for risks works very well. Tables typically summarise key points, rather than containing essential and detailed information. Perhaps it would be better to have the detailed text of the opportunities (based on the IASC categories), and then a table summarising the opportunities (by the IASC categories) and also a summary of the risks in the Table? The table structure can make clear that the risks cross-cut individual IASC categories.

For the text on risks, it feels that risk of various biases are not adequately addressed. While these may be generic AI risks for mental health, they may be even more magnified in crisis-affected populations. For example, automation bias (users accepting AI advice without critical reflection), negative interpretation bias (AI mirroring negative thought patterns), and confirmation bias (AI reinforcing rather than challenging negative or paranoid thought patterns).

Similarly, I’m not sure the issue of informed consent is adequately addressed. Lack of genuinely informed consent is a key issue among crisis-affected populations giving magnified power and dependency issues. This risk is likely magnified with the data collection underpinning AI systems. For example, see doi: 10.1186/s12910-025-01189-2.

Research agenda:

This is not really a research agenda as a research agenda would typically entail a strategic plan of long-term goals, priorities, and guiding principles, to act as a roadmap to guide decision-making and funding. Rather, what is presented is a list of research priorities. This is absolutely fine as they are still extremely useful, but it may just be good to use a more modest term than research agenda.

I wonder if it may also be helpful to explicitly include some key principles, including some that are already mentioned in the paper (but not explicitly referred to as principles). For example, supporting digital health equity, safety, epistemic justice and local ownership.

Related to the above point about this not really being a research agenda, perhaps a recommendation could be to set a research agenda by bringing together key global, national and local actors to establish consensus and legitimacy for such a research agenda (e.g. as done for MHPSS by the Tol et al. paper cited in the paper).

While digital inclusion is rightly mentioned for specific groups with physical, sensory or cognitive disabilities, shouldn’t the recommendations include addressing broader equity issues around digital connectivity. For example, economically/socially disadvantages groups withing crisis-affected populations as they are likely to have lower digital connectivity, and also inter-sectional issues around gender, race and ethnicity?

Environmental Sustainability and Resource Scarcity: Aren’t the energy and water demands associated with AI infrastructure (i.e. the huge data centres) located in higher-income countries, rather than in geographic areas of the users (i.e. crisis-affected countries)? I appreciate the issue raised but I’m not sure of its relevance to crisis-affected countries?

Responsible actors:

This section focuses much more on key principles/issues underpinning the research priorities, rather than the actors. Perhaps it should be re-named as key principles (see above)?

For funders, the Wellcome Trust could be mentioned given their commitment to funding mental health research. I think it’s also important to re-iterate the point that humanitarian crises create elevated and specific mental health needs and magnify critical AI-related issues (e.g. particular needs and issues – e.g. cultural calibration) and therefore dedicated research funding is required to support the effective, safe and equitable use of AI in crisis-affected contexts.

USAID no longer exists and so reference to it should be removed (p.6)

General:

Given the journal, the readers are mental health academics and practitioners and policy makers, not expert AI users. Therefore, it would be good to clarify some terms. E.g. LLM (no wording or explanation is provided). Similarly, what a ‘small language model’ consists of.

I appreciate there’s probably a jouranl limit on the number of references, but other relevant literature reviews could perhaps be cited? For example, doi.org/10.3389/frai.2025.1627773

Thank you for this paper which makes an important contribution to the field of MHPSS.

# Reviewer report: Review: The humanitarian AI paradox: Key opportunities, challenges and research needs for the use of AI in humanitarian mental health response — R0/PR4

**DOI:** 10.1017/gmh.2026.10254.pr4

Peer Review of Manuscript GMH-2026-0141 “The Humanitarian AI Paradox: Defining a Research Agenda for the Use of AI in Humanitarian Mental Health Response”

Overall Impression

This manuscript addresses a highly relevant and timely topic that warrants urgent discussion and is well suited for an academic audience interested in global mental health. I read the paper with great interest; it stimulates further reflection on complex issues that are not yet fully understood in the field. The topic is important, and the manuscript has clear potential to make a meaningful contribution. However, the paper appears somewhat hastily prepared and would benefit from further refinement before publication.

Major Comments

1. On page 2 (line 26), the authors indicate that they will discuss six concurrent trends; however, only four are presented. This inconsistency should be addressed. More broadly, the section describing these trends is interesting but could benefit from clearer structuring and more accessible language for non-specialist readers.

2. The manuscript refers to the “IASC pyramid”, which may not be immediately recognisable to all readers. It would be more precise and accessible to refer to this as the multi-layered intervention pyramid from the 2007 IASC Guidelines on Mental Health and Psychosocial Support. Clarifying this terminology would improve readability and inclusivity for a broader audience.

The discussion of risks associated with AI in MHPSS is compelling, particularly the identification of eight distinct risks. I feel the issue of misreading cultural expressions of distress deserves more in-depth treatment and it is actually than just ‘misreading’ . There is a real risk that the dominance of Anglo-American perspectives could lead to further “Americanisation” of how distress is conceptualised and addressed globally, which could amount to “epistemological violence”: forcing the whole world to think and speak as Americans do. The manuscript would benefit from a deeper exploration of this issue, including how AI systems might (inadvertently?) standardise or homogenise culturally diverse expressions of distress, paroptuclalry where expressions of distress are fluid and not well delineated. See for example Ventevogel et al. (2013). So my question is whether the authors could explore whether AI can be leveraged to avoid marginalisation and perhaps even to actively support diverse epistemologies. For example: Could AI tools be designed to help communities articulate distress in ways that align with their own cultural frameworks? Could participatory or co-designed AI systems enable communities to shape psychoeducational materials or conversational tools according to their own needs and conceptualisations? I am not sure this is easy to do, but it would be so great if communities including those those who do not have a strong written record for mental health language could use the tools to generate materials that they deem appropriate and fit. This is a purely optional suggestion but I would be interested if the AI tools could be use to the advance of marginalized groups and empower them rather than mold them into the dominate framework. I have not read much about this although the authors of a recent overview paper on digital mental health interventoons seem to allude this when they say that “Digital mental health interventions must include culturally and contextually relevant strategies that build trust and acknowledge the unique needs of these populations. This involves the creation of culturally competent materials, the provision of multilingual support and involving end users and community leaders in programme design and implementation.” (Househ et al., 2026)

Minor Comments

1. The manuscript would benefit from careful proofreading to address minor inconsistencies and improve overall clarity.

a. For example the capitalizing in reference 2 is incorrect.

b. Reference 22: the mhGAP-HIG is also authorerd by UNHCR.

c. On page 6, the authors call for long-term research support from USAID. Given recent institutional changes, this reference may no longer be appropriate or realistic. The authors should revise this point to reflect the current funding and policy landscape.

Conclusion

This is an important and thought-provoking manuscript with strong potential.

References

Househ, M., Shah, H. A., Tariq, Z. U. A., Alsayed Hassan, D., Khalifa, M., Schneider, J., Hamdi, M., Abd-Alrazaq, A., Ahmad, A., Solaiman, B., Kushniruk, A., & Al-Nuaimi, S. K. (2026). Digital mental health needs a purpose-driven approach. Nature Human Behaviour, 10(2), 227–238. https://doi.org/10.1038/s41562-025-02380-6

Ventevogel, P., Jordans, M., Reis, R., & de Jong, J. (2013). Madness or sadness? Local concepts of mental illness in four conflict-affected African communities. Conflict and Health, 7(1), 3. https://doi.org/10.1186/1752-1505-7-3

# Editor report: Recommendation: The humanitarian AI paradox: Key opportunities, challenges and research needs for the use of AI in humanitarian mental health response — R0/PR5

**DOI:** 10.1017/gmh.2026.10254.pr5

**Affiliations:** Psychology, University of Washington, United States

Thank you for your thought-provoking submission. I have reviewed the article as well as the comments from 3 expert reviewers. I agree with the reviewers that this article discusses an important and timely topic in the flied of global mental health.

In my own review, I think it would be helpful to revisit the introduction (particularly paragraph 2 and lead into paragraph 3) to ensure that you are making clear the connection back to mental health and psychosocial support within the context of humanitarian crisis response. As the paragraph currently reads, it sets up a need for AI oversight in humanitarian crisis response more generally.

# Editor report: Decision: The humanitarian AI paradox: Key opportunities, challenges and research needs for the use of AI in humanitarian mental health response — R0/PR6

**DOI:** 10.1017/gmh.2026.10254.pr6

**Affiliations:** Johns Hopkins University Bloomberg School of Public Health, United States

No accompanying comment.

# Reviewer report: Review: The humanitarian AI paradox: Key opportunities, challenges and research needs for the use of AI in humanitarian mental health response — R1/PR8

**DOI:** 10.1017/gmh.2026.10254.pr8

Thank you for making the requested revisions. I am happy with them all.

# Reviewer report: Review: The humanitarian AI paradox: Key opportunities, challenges and research needs for the use of AI in humanitarian mental health response — R1/PR9

**DOI:** 10.1017/gmh.2026.10254.pr9

Overall, I thank the authors for their considerable efforts in revising this manuscript. The paper has improved substantially and the authors have emagged with the extstive comments of the reviewers.

I have no major concerns and only a few minor discretionary suggestions for clarification.

On page 5, line 15, the term “hallucinations” may benefit from brief explanation given its different meaning in clinical mental health contexts.

On page 7, lines 9–11, the definition of prompt injection attacks could perhaps be expressed more clearly.

On page 8, in the third bullet point (lines 22–26), the authors may wish to explicitly mention the humanitarian MHPSS coordination mechanism through the IASC Reference Group as a potential actor in coordinating a research agenda for AI in MHPSS and humanitarian settings.

Table 2 which was added on request of another reviewer is helpful, but not essential in my view.

# Editor report: Recommendation: The humanitarian AI paradox: Key opportunities, challenges and research needs for the use of AI in humanitarian mental health response — R1/PR10

**DOI:** 10.1017/gmh.2026.10254.pr10

**Affiliations:** Psychology, University of Washington, United States

Well done attending to the reviewer’s comments. Reviewer 2 had some minor comments for further consideration. I do agree that adding some clarification could be helpful, particularly regarding “hallucinations” (given the overlap with psychiatric terminology).

# Editor report: Decision: The humanitarian AI paradox: Key opportunities, challenges and research needs for the use of AI in humanitarian mental health response — R1/PR11

**DOI:** 10.1017/gmh.2026.10254.pr11

**Affiliations:** Johns Hopkins University Bloomberg School of Public Health, United States

No accompanying comment.
